# Expanding Applications of Three-Dimensional Cardiac Mapping Systems: A Review

**DOI:** 10.3390/jcm14186487

**Published:** 2025-09-15

**Authors:** Rabeia Javid, Stephen O. Otieno, Stephen B. Wheatcroft, Sacchin Arockiam, Muzahir H. Tayebjee

**Affiliations:** 1 Leeds Institute of Cardiovascular and Metabolic Medicine, School of Medicine, University of Leeds, Leeds LS2 9JT, UK; rabeia.javid@nhs.net (R.J.); stephen.wheatcroft@nhs.net (S.B.W.); 2 Leeds General Infirmary, Department of Cardiology, Leeds Teaching Hospitals NHS Trust, Great George Street, Leeds LS1 3EX, UK; stephen.omondi@nhs.net (S.O.O.); sacchin.arockiam@nhs.net (S.A.)

**Keywords:** electro-anatomical mapping, percutaneous coronary intervention, contrast-induced nephropathy, radiation exposure, electrophysiology, cardiac device implantation

## Abstract

Percutaneous coronary intervention (PCI) is a widely performed revascularisation procedure for coronary artery disease. Although effective, its reliance on fluoroscopy and iodinated contrast exposes patients and operators to risks of radiation and nephrotoxicity. As PCI techniques have become more complex, interest has grown in imaging methods that reduce dependence on fluoroscopy and contrast. Electro-anatomical mapping systems (EAMS), developed for catheter navigation in electrophysiology, enable real-time three-dimensional visualisation without the need for fluoroscopy or contrast. By adapting coronary guidewires as electrodes, EAMS can reconstruct vessel anatomy and track interventional tools in real time. EAMS have demonstrated feasibility and safety in device implantation, and early studies suggest their applicability to PCI, where they may mitigate radiation and contrast exposure by providing an alternative method for guidewire and stent visualisation. This review provides a narrative overview of current evidence, outlining the technical principles, applications in device implantation, and the emerging role of EAMS in coronary intervention.

## 1. Introduction

Percutaneous coronary intervention (PCI) remains the principal method of revascularisation for patients with symptomatic coronary artery disease, with millions of procedures performed globally each year [1]. However, its reliance on fluoroscopy and iodinated contrast presents important limitations, including cumulative radiation exposure for patients and operators [2] and the risk of contrast-induced nephropathy (CIN) in susceptible groups [3,4]. Adjunctive imaging tools such as intravascular ultrasound (IVUS), optical coherence tomography (OCT), and dynamic coronary roadmap (DCR) software can reduce contrast use while improving procedural precision [5,6,7], but all remain dependent on fluoroscopic platforms and typically require some contrast administration.

Electro-anatomical mapping systems (EAMS), developed for catheter ablation in electrophysiology [8], offer an alternative approach—enabling three-dimensional navigation within cardiac chambers without the need for fluoroscopy or contrast. They can support near-zero fluoroscopy procedures, substantially reducing radiation exposure for both patients and operators [9]. Their use has been investigated in cardiac device implantation, with early studies reporting favourable outcomes in terms of safety, anatomical accuracy, and procedural feasibility in selected patients [10,11]. More recently, case reports and feasibility studies have described their potential application in coronary intervention [12,13]. This review brings together these emerging reports to provide a narrative overview of current evidence, outlining the technical principles of EAMS, their investigational use in device implantation, and the emerging evidence supporting their role in PCI. We include representative studies identified through author expertise and targeted literature exploration, recognising that the available evidence base remains small and exploratory.

## 2. Technical Foundations of Electro-Anatomical Mapping Systems

EAMS are fundamental tools in electrophysiology, generating real-time three-dimensional (3D) reconstructions of cardiac chambers. Using adhesive patches placed on the patient’s chest, the system establishes an electrical or magnetic reference field that continuously tracks catheter positions within a defined coordinate framework [8]. Spatial and electrical data are integrated to produce detailed anatomical maps, which provide the basis for accurate arrhythmia diagnosis, chamber navigation with minimal fluoroscopy, and arrhythmia substrate ablation [9].

As the operator manoeuvres the catheter through cardiac chambers, the system records its location as x, y, z coordinates. Thousands of these points are accumulated to create a dense point cloud that accurately captures chamber contours and geometry. Modern systems incorporate motion compensation using gating algorithms and reference sensors to maintain positional accuracy despite respiratory and cardiac motion [14]. Current technology achieves a spatial resolution of 1–2 mm, generating surface models that can be manipulated in real time. These maps delineate chamber geometry and surface contours but do not provide information about tissue depth or wall composition [14,15].

Building on these core mapping principles, EAMS are increasingly being explored in other interventional procedures. Their application in device implantation and coronary intervention adapts the same localisation technologies to new anatomical targets. The following sections examine how these systems are integrated into each workflow and summarise the current evidence supporting their feasibility.

## 3. EAMS in Device Implantation

Transvenous implantation of cardiac devices is conventionally performed under fluoroscopic guidance, with lead advancement and positioning guided by two-dimensional X-ray landmarks and often supplemented by contrast venography. As device complexity has increased—particularly with CRT—both radiation and contrast exposure have risen for patients and operators [16]. Procedural challenges are further compounded by anatomical variability, especially in the coronary sinus and venous system [17,18].

A key limitation of fluoroscopy is its inability to provide electrical or three-dimensional anatomical information, which is critical for optimising lead placement—especially the left ventricular (LV) lead in CRT. EAMS address this by enabling real-time 3D visualisation of cardiac chambers and catheters. Operators can reconstruct venous anatomy, identify scarred myocardium, and guide lead positioning with greater precision. These benefits have been demonstrated across a range of indications, including CRT, His bundle pacing (HBP), and left bundle branch pacing (LBBP). Illustrative case reports are summarised in Table 1, while larger prospective and retrospective series are presented in Table 2.

HBP and LBBP aim to preserve physiological ventricular activation by directly engaging the His–Purkinje system. These techniques are technically demanding and often associated with prolonged fluoroscopy exposure. EAMS facilitate non-fluoroscopic localisation of the conduction system, improving procedural accuracy and reducing radiation dose.

Multiple prospective and retrospective studies have shown that EAMS-guided implantation can significantly reduce fluoroscopy time and contrast use (Table 2). While most reported cases have used the EnSite system, CARTO, Rhythmia, and Kodex EPD platforms are feasible. A key technical distinction is that EnSite allows the pacing lead to serve as a mapping electrode, whereas CARTO typically requires a dedicated navigation catheter to establish geometry [42].

The integration of EAMS into device implantation workflows enables precise anatomical mapping and real-time catheter tracking while reducing reliance on radiation and contrast media. Further studies are needed to clarify long-term outcomes, cost-effectiveness, and training requirements before widespread adoption in routine clinical practice.

## 4. EAMS in Coronary Intervention

### 4.1. Imaging Technologies Currently Used to Support PCI

Conventional PCI relies primarily on fluoroscopy and iodinated contrast media to visualise coronary anatomy and guide interventional tools. While this approach remains the standard of care, both radiation exposure and contrast use present important limitations—particularly in complex interventions and in patients with comorbidities [3,4,50].

**Figure 1:**
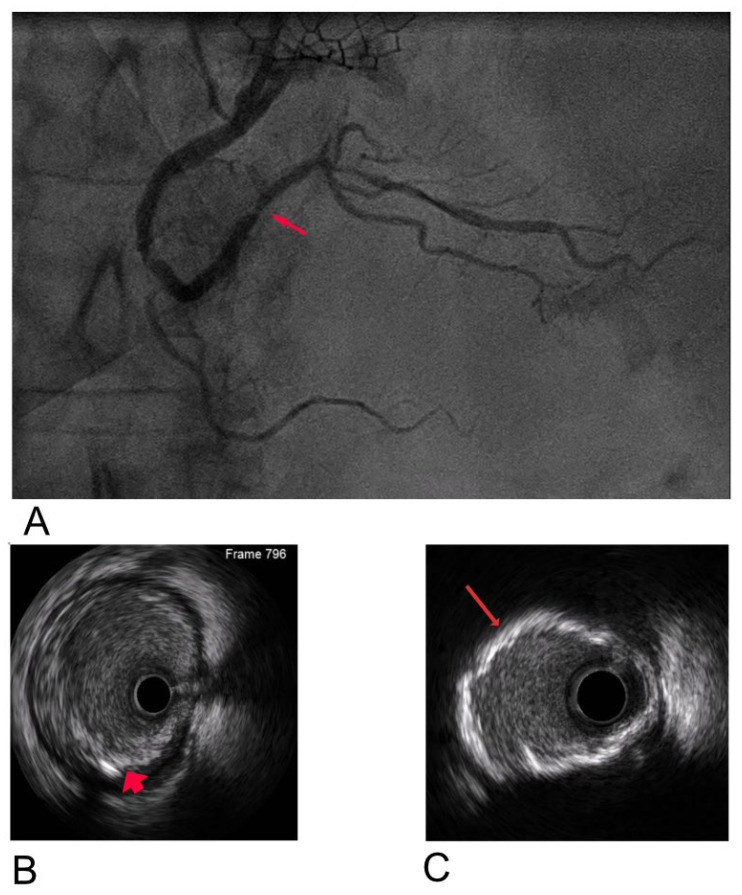
Multi-plane illustration of right coronary artery (RCA) assessment: (**A**) Coronary angiogram showing multiple areas of stenosis, with one marked by an arrow. (**B**) IVUS image demonstrating a fibrocalcific plaque in the mid RCA (arrow). (**C**) IVUS image showing a 360° arc of calcium in the RCA, with the arrow highlighting the calcification.

**Figure 2:**
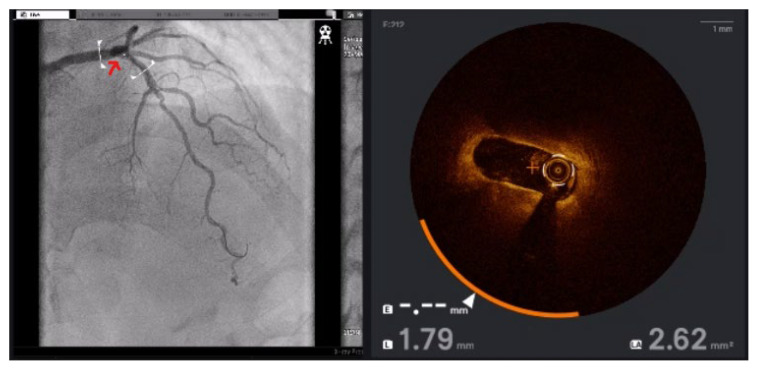
OCT of the left anterior descending (LAD) artery. Red arrow indicates the referenced OCT segment correlating with the angiogram. OCT reveals severe proximal LAD stenosis not readily apparent on angiography.

**Figure 3:**
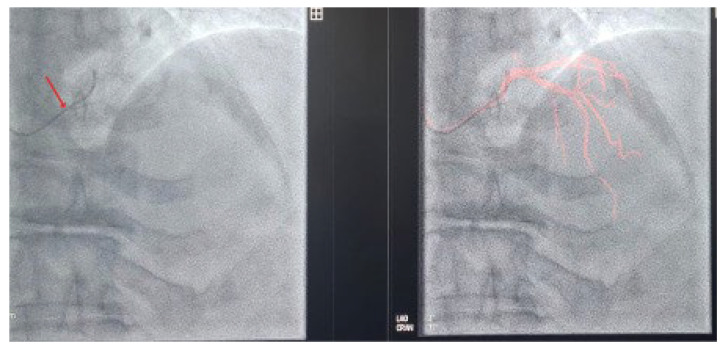
JL 3.5 coronary catheter (arrow) positioned in the left coronary cusp in LAO cranial view following coronary angiography (**left**). DCR map visualising the coronary tree without contrast injection (**right**).

To address these challenges, adjunctive imaging modalities have been adapted to enable ultra-low contrast PCI. Intravascular ultrasound (IVUS) provides detailed cross-sectional images without reliance on contrast media (Figure 1) and is recommended in complex interventions [5,6]. OCT provides high-resolution plaque characterisation and stent assessment (Figure 2), but requires flushing, typically with contrast media; however, substitutes such as low-molecular-weight dextran and heparinised saline have shown promise as alternatives [51,52,53,54]. DCR software overlays a live coronary roadmap onto the fluoroscopic image, enabling navigation of coronary tools and stent delivery without repeated contrast injections (Figure 3) [7,55]. While these modalities provide valuable anatomical information, they remain dependent on fluoroscopic platforms and do not eliminate contrast use entirely, highlighting the potential role for complementary navigation technologies such as EAMS.

### 4.2. Technical Principles and Setup of EAMS in PCI

EAMS have already been used in electrophysiology to track coronary anatomy. During epicardial ventricular tachycardia and premature ventricular contraction ablation procedures, angioplasty guidewires have been employed as mapping electrodes to delineate the course of the coronary arteries and reduce the risk of ablation-related injury [56,57,58,59].

Building on this concept, EAMS in PCI can track insulated angioplasty guidewires using the same localisation technologies—impedance fields, magnetic sensors, or hybrid systems [14,15]. By selectively insulating the guidewire shaft and leaving only the distal tip exposed, the mapping system registers three-dimensional positional data from the tip as it traverses coronary vessels, thereby reconstructing vessel geometry. This map can then guide interventions such as stent delivery without the need for X-ray or contrast.

Successful localisation depends critically on consistent insulation and current density. Variations in guidewire preparation can affect positional accuracy, and the precise sensing location may vary subtly across customised wires. While standard coronary guidewires are not designed for EAMS integration, experimental setups have demonstrated reliable tracking using modified wires. At present, no guidewires are commercially approved for this application. The Vision wire (Biotronik, Berlin, Germany), for example, is insulated but not licensed for PCI due to safety concerns.

Although the core localisation principles of EAMS remain consistent, mapping coronary vessels poses distinct technical challenges compared with endocardial chamber reconstruction. The coronary lumen is narrow, tortuous, and continuously moving, with less catheter support and a higher risk of wall trauma. Unlike the relatively stable intracavitary environment, coronary mapping is more susceptible to motion artefact and contact variability. Existing mapping algorithms—such as respiratory gating and point filtering—may require adjustment to maintain fidelity in this setting. These factors underscore the need for procedural adaptations and tool optimisation when translating EAMS to coronary interventions.

### 4.3. Feasibility Studies of EAMS in PCI

The first reported human case used the EnSite NavX system to guide PCI in a patient with advanced renal impairment [12]. An angioplasty guidewire was configured as an electrode using the insulation technique described above. This enabled the system to reconstruct the right coronary artery (RCA) in three dimensions, identify a mid-vessel culprit lesion, and localise the wire tip in real time within the coronary lumen via an impedance field between the guidewire and surface patches. The lesion was then confirmed with minimal fluoroscopy and contrast, and stenting was successfully completed with the same mapping-assisted navigation technique. No adverse events occurred.

An experimental porcine model further demonstrated the feasibility of EAMS-guided PCI [60]. Vascular anatomy was reconstructed from computed tomographic angiography (CTA) and registered to the electro-anatomical workspace using Verismo software. Real-time tracking was achieved with an impedance-based navigation system after integrating these CT-derived models. Balloon-expandable stents and guidewires were modified to include embedded electrodes, allowing catheter navigation without fluoroscopy or contrast. Interventions were performed in both coronary and carotid arteries using predefined virtual lesions. Procedural outcomes—including stent positioning, deployment accuracy, and apposition—were validated using OCT and, when necessary, confirmatory fluoroscopy. The approach achieved a localisation accuracy of 90.9% and a precision of 1.4 mm, with successful stent deployment in 82% of attempts. OCT with a saline flush confirmed complete stent wall apposition in the coronary arteries. This study demonstrated that electrode-equipped PCI hardware can be tracked reliably within an EAMS environment, enabling real-time three-dimensional guidance in the absence of radiation and contrast.

**Figure 4:**
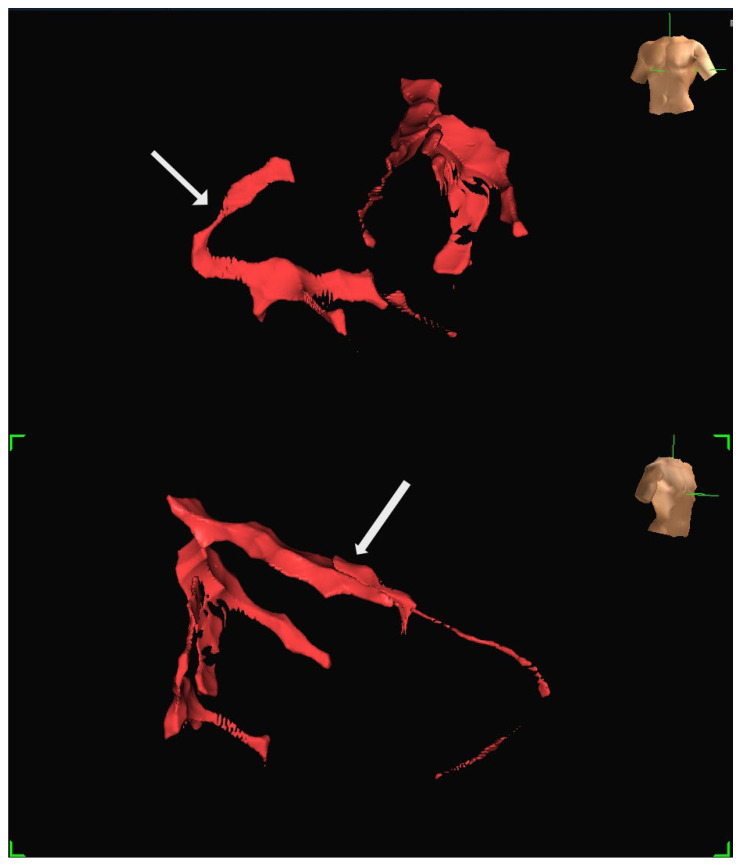
Representative three-dimensional reconstruction of coronary anatomy generated using the EnSite Precision EAMS. The geometry was derived from guidewire tracking in a single patient. The coronary vessels are displayed in red: (**Top**) Left anterior oblique (LAO) projection, with the RCA marked by an arrow. (**Bottom**) Right anterior oblique projection (RAO), with LAD marked by an arrow. Torso icons indicate image orientation.

**Figure 5:**
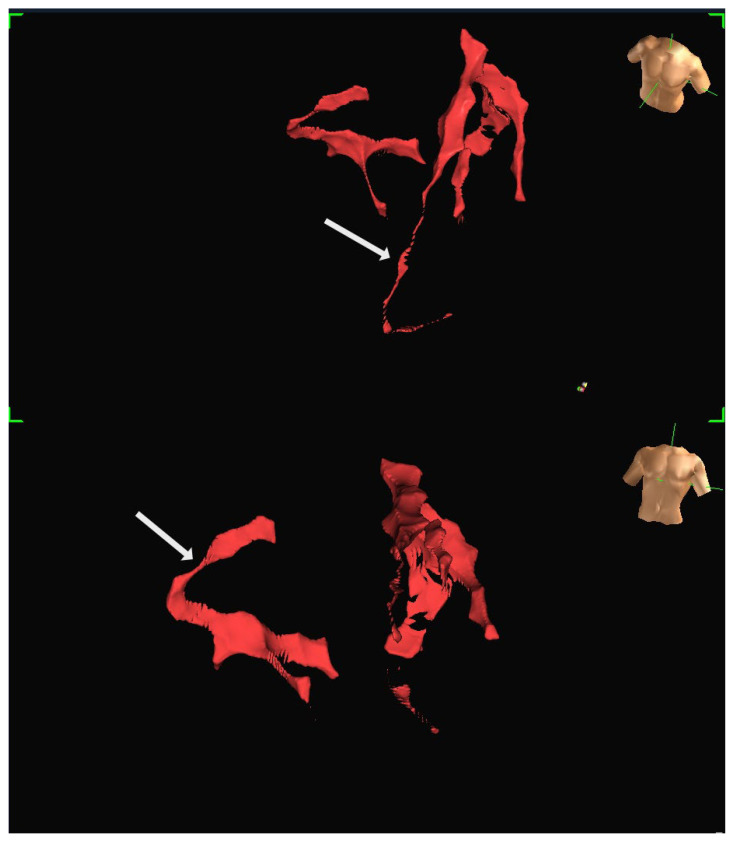
Additional views of the same patient dataset shown in Figure 4, demonstrating the ability to visualise the vessel course from different angles: (**Top**) The LAD is marked by an arrow. (**Bottom**) RCA is marked by an arrow. Both images are displayed in LAO projection at different angulations.

A human feasibility study involving multiple participants was conducted using the EnSite Precision system [13]. Insulated Sion Blue coronary guidewires (Asahi Intec, Japan) were tracked through epicardial arteries, including the left anterior descending and right coronary arteries, to produce anatomical reconstructions closely matching conventional angiographic images. The mapping system tracked the distal wire tip based on impedance field localisation. System accuracy was further evaluated in a custom-built water bath model incorporating segmented CT data. In this controlled setup, bipolar catheters fabricated from over-the-wire balloons and coronary wires were advanced through fixed vessel phantoms, confirming consistent localisation and map generation. Representative three-dimensional reconstructions generated using the EnSite Precision mapping system are shown in Figure 4 and Figure 5. These panels depict different views from the same patient, illustrating how coronary anatomy was mapped and visualised during the feasibility study.

Collectively, these studies demonstrate the technical feasibility of EAMS for PCI. Early investigations have shown that coronary anatomy can be reconstructed, and stent delivery guided with minimal reliance on conventional imaging. Although current approaches require customised guidewires and complex registration, they provide a compelling proof-of-concept for contrast- and fluoroscopy-sparing coronary intervention. A summary of findings is provided in Table 3. Further development of mapping-compatible tools and streamlined workflows will be essential for broader clinical translation.

### 4.4. Limitations and Future Directions for EAMS in PCI

Although early feasibility studies are encouraging, EAMS-guided PCI remains an investigational approach with several important limitations that must be acknowledged.

A central limitation is the relatively low spatial resolution compared with intravascular imaging. While OCT achieves axial resolution of 10–15 μm and IVUS 100–200 μm, current EAMS offer localisation accuracy in the millimetre range. This restricts the ability of EAMS to provide fine morphological detail, particularly in complex or calcified lesions.

IVUS and OCT already enable minimal or no-contrast PCI while providing superior diagnostic detail. EAMS cannot resolve critical procedural findings such as plaque morphology, stent expansion, minor dissections, or intramural haematomas—findings readily detectable with IVUS and OCT [61,62]. These features are strongly linked to both acute procedural success and long-term prognosis following PCI [63,64].

Accuracy is further influenced by coronary motion, respiration, and field drift, which may result in small but clinically relevant localisation errors. Spatial accuracy is also influenced by non-standard electrode size and geometry, which affect the stability of localisation signals and introduce uncertainty into map generation. In addition, the underlying localisation technology can affect precision: impedance-based systems are more susceptible to field distortion and drift, whereas magnetic systems may offer greater positional stability but require specialised hardware.

There are no commercially available guidewires designed for mapping integration, necessitating the custom insulation of standard wires. This preparation is technically demanding and influences contact stability and localisation fidelity.

The coronary vasculature poses unique mapping challenges compared with intracardiac chambers, including smaller diameters, tortuous geometry, and continuous motion. These factors can contribute to signal dropout, reduced map density, and catheter instability. Safety considerations—such as the risk of coronary spasm, dissection, or trauma from stiff or insulated guidewires—also require careful attention in future studies.

The cost-effectiveness of EAMS-guided PCI remains uncertain. Mapping systems and disposable components incur significant upfront costs. However, these may be offset by reductions in contrast-related complications, radiation exposure, and downstream healthcare utilisation—particularly in high-risk patients. Formal health economic analyses are needed to quantify potential savings, especially when factoring in avoided hospitalisations for contrast-induced nephropathy or cumulative radiation risks to staff. Cost–benefit modelling should be incorporated into future prospective studies.

Compared with established low-contrast PCI approaches, such as IVUS- or OCT-guided strategies, EAMS adds unique value by enabling three-dimensional navigation without reliance on fluoroscopy or contrast. While intravascular imaging remains the gold standard for lesion assessment and stent optimisation, EAMS may serve as a complementary tool that reduces incremental contrast and radiation exposure. Hybrid workflows—in which EAMS facilitates guidewire navigation and device delivery while IVUS or OCT ensures high-resolution assessment—may provide the safest and most effective path toward near-zero fluoroscopy PCI. This role may be particularly relevant in patients with advanced renal impairment or when conventional imaging strategies are contraindicated.

Future research should prioritise the development of dedicated coronary mapping tools optimised for EAMS integration. These may evolve beyond single-electrode guidewires to incorporate multi-electrode designs, which could accelerate vessel reconstruction and improve localisation fidelity. Validation through large clinical trials will be necessary to establish procedural safety, reproducibility, and clinical benefit. Continued innovation in mapping technology—building on successful anatomical reconstructions with 3D EAMS—may ultimately support near-zero fluoroscopy PCI with minimal contrast use. This approach holds particular promise for patients with advanced renal impairment or when conventional imaging strategies are contraindicated.

## 5. Conclusions

While EAMS-guided PCI is not yet part of routine clinical practice, its potential to minimise contrast and radiation exposure may benefit carefully selected patient populations—particularly those with advanced renal impairment or contraindications to conventional imaging. However, the long-term clinical significance of reducing radiation in PCI remains uncertain, as typical exposures are modest relative to harmful thresholds [65]. Accordingly, this benefit should be interpreted cautiously. As mapping technologies evolve, interventional cardiologists should recognise that EAMS are most likely to be complementary rather than substitutive, with EAMS supporting navigation and intravascular imaging, ensuring high-resolution lesion assessment and stent optimisation. To establish EAMS-guided PCI in routine practice, further innovation and validation are needed, including the development of dedicated coronary mapping tools, integration with imaging modalities such as IVUS, OCT, CT, or MRI, and multicentre clinical trials to assess outcomes and cost-effectiveness.

## Figures and Tables

**Table 1:** Case reports of EAMS in device implantation.

Case Report	*n*	Gender	Indication	Device	EAMS	Catheter for Anatomy	Time (min)	Procedure Time	Complication	Fluoroscopy Time	Radiation Exposure
Kloosterman 2000 [19]	1	Female	AVN ablation	Dual chamber PM	CARTO	–	10	-	None	n/s	n/s
Ruiz-Granell 2005 [20]	1	Male	AVN ablation	Single-chamber PM	EnSite NavX	7F 4 mm tip quadripolar steerable ablation catheter	n/s	-	None	<1 min	Minimal
Merino 2008 [21]	1	Female, 24 weeks pregnant	VT and SSS	Dual chamber ICD	EnSite NavX	Mapping catheter	n/s	180 min	None	0	0
Tuzcu 2007 [9]	1	Female, 21 weeks pregnant	VT	Single-chamber ICD	EnSite NavX	Single Deflectable quadripolar	n/s	120 min	None	0	0
Singh 2012 [22]	1	Male	Sinus node dysfunction and VT	Dual chamber ICD	EnSite	Mapping catheter	n/s	n/s	None	n/s	n/s
Velasco 2013 [23]	1	Female, 31 weeks pregnant	High-degree AV block	Dual chamber PM	EnSite (NavX)	Quadripolar catheter	n/s	n/s	None	<5 s	n/a
Kühne 2015 [24]	1	Female, 9 weeks pregnant	AV block	Single-chamber PM	CARTO 3	Mapping catheter	n/s	n/s	None	0	0
Hartz 2017 [25]	1	Female, pregnant	Sinus node dysfunction,	Single-chamber ICD	CARTO 3	Deflectable mapping catheter	n/s	n/s	None	0.5 s	n/a
Payne 2017 [26]	1	Female, 11 weeks pregnant	PAF	Dual chamber PM	EnSite NavX	Single Deflectable quadripolar	n/s	n/s	None	<10 s	<1 mGy
Ringwala 2017 [27]	1	Male	AVN ablation and His bundle pacing	Single chamber PM with HBP	EnSite NavX	Octapolar catheter	n/s	n/s	Healthcare-associated pneumonia	n/a	n/s
Cay 2018 [28]	1	Male	Symptomatic AV block	Dual chamber PM	n/s	Steerable decapolar + quadripolar	n/s	n/s	None	Yes (n/s)	Yes (n/s)
Paech 2019 [29]	1	n/s	n/s	–	EnSite Precision	Steerable decapolar catheter	n/s	n/s	None	–	49.2 cGy/cm^2^
Hua 2019 [30]	1	Male	Sinus node dysfunction	Dual chamber PM with HBP	KODEX-EPD	His pacing lead	n/s	n/s	None	n/s	n/s
Molina-Lerma 2020 [31]	1	Female	Advanced AVB	Dual chamber PM with LBBP	EnSite + ICE	Tendril STS 52 cm STS; Abbott	n/s	n/s	None	n/s	–
Žižek 2021 [32]	1	Female	Advanced AVB	Dual chamber PM	EnSite Precision	10-polar Polaris X	n/a	n/s	None	3 min	n/a
Covino 2021 [33]	1	Female	AF refractory to drug therapy	Single-chamber HBP	Rhythmia HDX	Intellamap Orion	-	75	None	13.6 min	16.40 Gy·cm^2^

Abbreviations: AVN, atrioventricular node; VT, ventricular tachycardia; SSS, sick sinus syndrome; AV, atrioventricular; AVB, atrioventricular block; PAF, paroxysmal atrial fibrillation; HBP, His bundle pacing; LBBP, left bundle branch pacing; PM, pacemaker; ICD, implantable cardioverter defibrillator; ICE, intracardiac echocardiography; AF, atrial fibrillation; EAMS, electro-anatomical mapping; n/s, not specified; n/a, not applicable.

**Table 2:** Prospective and retrospective studies utilising EAMS for pacing and CRT implantation (including LBBP, HBP).

Case Report/Study	Study Type	*n*	Participants	Indication	Device	EAMS	Complications
Ruiz-Granell 2008 [34]	Retrospective case-control	15	Human	AV block	Single-chamber PM	EnSite NavX	1:Lead dislodgement in EAMS arm (6.6%)
Choudhuri 2009 [35]	Prospective, no control	7	Dog	Normal	Single chamber	Electromagnetic Navigation	1:Lead dislodgement in EMN arm (14%)
Del Greco 2012 [36]	Prospective	4	Human	NYHA Class IV, EF < 35%	CRT-ICD	EnSite NavX	None
Castrejón 2013 [37]	Prospective	35	Human	Primary and secondary prevention	Single and dual chamber ICD	EnSite NavX	Infection (2.8%)
Mina 2013 [38]	Retrospective control	21	Human	NICMP, ICMP	CRT-D = 19, CRT-P = 2	EnSite Velocity	Pneumothorax in control
Mafi Rad 2015 [39]	Prospective	25	Human	LBBB	CRT-D = 23, CRT-P = 2	EnSite NavX	None
Del Greco 2017 [10]	Prospective	125	Human	NICMP, ICMP	CRT	EnSite	CS dissection, Pocket hematoma, Pocket infection
Colella 2016 [40]	Prospective case-control	61	Human	NICMP, ICMP	CRT	EnSite Velocity	None
Guo 2018 [41]	Prospective case-control	Case 6, Control 21	Human	NICMP, ICMP	Single and dual chamber	EnSite NavX	None
Huang 2019 [42]	Prospective case-control	20	Human	NICMP, ICMP	CRT	EnSite NavX, CARTO 3	None
Patel 2019 [43]	Prospective case-control	33	Human	Not stated	Single and dual chamber ICD	EnSite NavX	None
Larsen 2019 [44]	Prospective case-control	140	Human	Not stated	Single, dual, biventricular	EnSite Velocity	None
Vijayaraman 2019 [45]	Prospective case series	3	Human	AVB	Dual chamber, AVN, LBBP	EnSite Precision	None
Sharma 2019 [46]	Prospective	10	Human	AVB, SND	Dual chamber HBP	CARTO	1 high threshold
Imnadze 2020 [47]	Prospective	15	Human	AVB	Dual chamber HBP	EnSite Precision	None
Sun 2020 [48]	Prospective	18	Human	AVB	AVN ablation + HBP	CARTO 3	None
Richter 2021 [11]	Prospective	58	Human	AVB, SND	Dual chamber, CRT-P, CRT-D	EnSite Precision	None
Richter 2023 [49]	Prospective	32	Human	AVB, SND	Dual chamber CRT	EnSite Precision	Hematoma, Pneumothorax

Abbreviations: CARTO, localises only special catheters; ICMP, ischaemic cardiomyopathy; NICMP, non-ischaemic cardiomyopathy; AVB, atrioventricular block; SND, sinus node dysfunction; HBP, His bundle pacing; LBBP, left bundle branch pacing; CRT, cardiac resynchronisation therapy; PM, pacemaker; ICD, implantable cardioverter defibrillator; CS, coronary sinus; EAMS, electro-anatomical mapping.

**Table 3:** Summary of feasibility studies exploring EAMS use in PCI.

Study	Setting	*n*	System Used	Key Finding	Limitation
Nair et al. [12]	Human case report	1	EnSite NavX	Successful PCI with EAMS in renal impairment	Customised insulated guidewire needed
Dorval et al. [60]	Porcine model (animal)	–	EnSite Velocity	Accurate coronary anatomy reconstruction with EAMS	Experimental model only
Javid et al. [13]	Human feasibility study	5	EnSite Precision	Feasibility of coronary mapping and tracking validated	Custom-built equipment used

Abbreviations: EAMS: Electro-anatomical mapping systems; PCI: Percutaneous coronary intervention.

## Data Availability

No new data were created or analysed in this study. Data sharing is not applicable to this article.

## Abbreviations

The following abbreviations are used in this manuscript:

PCI	Percutaneous Coronary Intervention
EAMS	Electro-Anatomical Mapping Systems
CIN	Contrast-Induced Nephropathy
IVUS	Intravascular Ultrasound
OCT	Optical Coherence Tomography
DCR	Dynamic Coronary Roadmapping
CRT	Cardiac Resynchronisation Therapy
HBP	His Bundle Pacing
LBBP	Left Bundle Branch Pacing
VT	Ventricular Tachycardia
